# Tunable phonon blockade in quadratically coupled optomechanical systems

**DOI:** 10.1038/s41598-018-20568-x

**Published:** 2018-02-02

**Authors:** Hai-Quan Shi, Xiao-Tong Zhou, Xun-Wei Xu, Nian-Hua Liu

**Affiliations:** 1 School of Materials Science and Engineering, Nanchang University, Nanchang, 330031 China; 2 Department of Applied Physics, East China Jiaotong University, Nanchang, 330013 China; 3 Institute for Advanced Study, Nanchang University, Nanchang, 330031 China

## Abstract

We theoretically investigate the phonon statistics of a quadratically coupled optomechanical system, in which an effective second-order nonlinear interaction between an optical mode and a mechanical mode is induced by a strong optical driving field on two-phonon red-sideband resonance. We show that strong phonon antibunching can be observed even if the strength of the effective second-order nonlinear interaction is much weaker than the decay rates of the system, by driving the optical and mechanical modes with weak driving fields respectively. Moreover, the phonon statistics can be dynamically controlled by tuning the strengths and the phase difference of the weak driving fields. The scheme proposed here can be used to realize tunable single-phonon sources with quadratically optomechanical coupling.

## Introduction

Phonon blockade^1^, in analogy to the Coulomb blockade^2^, photon blockade^3^ and Rydberg blockaded^4–8^, is a quantum phenomenon that only one phonon can be excited in a nonlinear mechanical oscillator when it is driven by external fields. Phonon blockade has already been studied in a mechanical resonator coupled to a superconducting qubit in the dispersive^1,9–11^ and resonant^12,13^ regimes. Effective phonon-phonon interactions can be induced by the qubit and strong phonon antibunching effect can be observed for large coupling strength and moderate detuning between the mechanical resonator and the qubit.

In the past decades, optomechanical systems have drew great attention in researches on the foundations of quantum theory and quantum information processing (for reviews, see refs^14–18^.). Recently, two different groups studied phonon statistics in quadratically coupled optomechanical systems^19,20^. Seok and Wright found that antibunched single-phonon field appears for large optomechanical cooperativity^19^. Hong Xie *et al*. found that strong effective phonon-phonon nonlinear interaction as well as phonon blockade can be induced by a strong optical driving field in the quadratically coupled optomechanical system^20^.

In contrast to refs^1,9–12,19,20^, where the phonon blockade is induced by strong effective phonon-phonon interactions, an interference-based phonon blockade called unconventional phonon blockade (UCPNB) was studied in ref.^13^. UCPNB, due to the destructive interference between different paths for two-phonon excitation, can be obtained with weak effective phonon-phonon interactions is similar to the unconventional photon blockade in a weakly nonlinear system of photonic molecule^21–33^. More recently, UCPNB was studies in a coupled nonlinear mechanical system with weak nonlinearity^34^.

In this paper, we shall theoretically investigate UCPNB in a quadratically coupled optomechanical system. An effective second-order nonlinear interaction between an optical mode and a mechanical mode can be induced when the quadratically coupled optomechanical system is driven by a strong optical driving field on two-phonon red-sideband resonance. Beside the strong optical driving field, the optical and mechanical modes are also driven by a weak optical and mechanical fields respectively. Different from the previous studies^19,20^, we will show that strong phonon antibunching can be observed even if the strength of the effective second-order nonlinear interaction is much weaker than the decay rates of the system. Moreover, the phonon statistics can be dynamically controlled by tuning the strengths and the phase difference of the weak driving fields. The proposal provides a simple way to realize tunable single-phonon sources with quadratically optomechanical coupling.

## Results

### Theoretical model and analytical results

**Figure 1:**
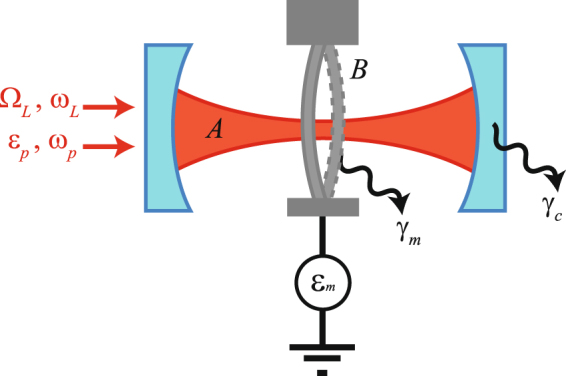
The schematic sketch of a quadratically coupled optomechanical system.

We study a quadratically coupled optomechanical system in which an optical mode is coupled to the second order of the position of a mechanical mode, as schematically shown in Fig. 1. The optical mode with frequency *ω*_*c*_ is driven by a strong driving field with the strength $|{{\rm{\Omega }}}_{L}|\gg \{{\gamma }_{c},{\gamma }_{m}\}$ and frequency *ω*_*L*_, where *γ*_*c*_ and *γ*_*m*_ are the damping rates of the optical and mechanical modes and Δ_*c*_ ≡ *ω*_*c*_ − *ω*_*L*_ is the frequency detuning between the strong driving field and the optical mode. Meanwhile, the optical mode and mechanical mode (frequency *ω*_*m*_) are driven by weak external fields with strengths {|*ε*_*p*_|, |*ε*_*m*_|} < {*γ*_*c*_, *γ*_*m*_} and frequencies {*ω*_*p*_, *ω*_*d*_}, with the detuning between the optical driving fields *δ*_*p*_ = *ω*_*p*_ − *ω*_*L*_. The Hamiltonian for quadratically coupled optomechanical system in the rotating reference frame with optical frequency *ω*_*L*_ takes the form (*ħ* = 1)

1 $$\begin{array}{rcl}H & = & {{\rm{\Delta }}}_{c}{A}^{\dagger }A+{\omega }_{m}{B}^{\dagger }B+g{A}^{\dagger }A{({B}^{\dagger }+B)}^{2}\\  &  & +({{\rm{\Omega }}}_{L}{A}^{\dagger }+{\varepsilon }_{p}{e}^{-i{\delta }_{p}t}{A}^{\dagger }+{\varepsilon }_{m}{e}^{-i{\omega }_{d}t}{B}^{\dagger }+{\rm{H}}.{\rm{c}}.),\end{array}$$

where *A* and *A*^†^ (*B* and *B*^†^) denote the annihilation and creation operators for the optical mode (mechanical mode), *g* > 0 describes the strength of the quadratic optomechanical coupling between the optical and mechanical modes, and H.c. stands for Hermitian conjugate. The quadratically optomechanical coupling can be found in the optomechanical crystals^35^, Fabry-Perot cavities with membrane-in-the-middle^36–39^, and some other optomechanical systems^40–43^.

The operators can be rewritten as the sum of their steady-state mean values and quantum fluctuation operators as: *A* → *α* + *a* and *B* → *β* + *b*, where *α* and *β* are the steady-state mean values, *a* and *b* are the quantum flucturation operators. The steady-state mean values *α* and *β* can be obtained approximately by setting the strength of the weak driving fields as zero, i.e. *ε*_*m*_ = *ε*_*p*_ = 0, then we have

2 $$\alpha =\frac{-i2{{\rm{\Omega }}}_{L}}{{\gamma }_{c}+i2{{\rm{\Delta }}}_{c}},$$

3 $$\beta =0.$$

After some standard procedures for operator linearization, the Hamiltonian for the quantum flucturation operators reads

4 $$\begin{array}{rcl}H^{\prime}  & = & {{\rm{\Delta }}}_{c}{a}^{\dagger }a+{\omega }_{m}{b}^{\dagger }b+g({|\alpha |}^{2}+{a}^{\dagger }a){({b}^{\dagger }+b)}^{2}\\  &  & +g(\alpha {a}^{\dagger }+{\alpha }^{\ast }a){({b}^{\dagger }+b)}^{2}\\  &  & +({\varepsilon }_{p}{e}^{-i{\delta }_{p}t}{a}^{\dagger }+{\varepsilon }_{m}{e}^{-i{\omega }_{d}t}{b}^{\dagger }+{\rm{H}}.{\rm{c}}.).\end{array}$$

For a strong optical driving field $|{{\rm{\Omega }}}_{L}|\gg \{{\gamma }_{c},{\gamma }_{m}\}$, we assume that the steady-state mean value *α* is much larger than the quantum flucturation operators *a* as ${|\alpha |}^{2}\gg \langle {a}^{\dagger }a\rangle$, so the term *ga*^†^*a*(*b*^†^ + *b*)^2^ in the above equation can be neglected. In the rotating reference frame with respect to the unitary operator *R*(*t*) = exp(*iδ*_*p*_*a*^†^*at* + *iω*_*d*_*b*^†^*bt*), the effective Hamiltonian can be obtained under the rotating-wave approximation by neglecting the terms oscillating with high frequencies in equation (4), e.g. 2*ω*_*d*_ and *δ*_*p*_ + 2*ω*_*d*_, as

5 $$\begin{array}{rcl}{H^{\prime} }_{{\rm{eff}}} & = & {{\rm{\Delta }}}_{p}{a}^{\dagger }a+{{\rm{\Delta }}}_{m}{b}^{\dagger }b+J({e}^{-i\theta }{e}^{-i\delta t}a{b}^{\dagger 2}+{e}^{i\theta }{e}^{i\delta t}{a}^{\dagger }{b}^{2})\\  &  & +({\varepsilon }_{p}{a}^{\dagger }+{\varepsilon }_{m}{b}^{\dagger }+{\rm{H}}{\rm{.c}}.),\end{array}$$

where the detunings *δ* = *δ*_*p*_ − 2*ω*_*d*_, Δ_*p*_ = Δ_*c*_ − *δ*_*p*_, Δ_*m*_ = *ω*_*m*_ + 2*g*|*α*|^2^ + *g* − *ω*_*d*_, and we assume that the detunings satisfy the condition $\{|\delta |,|{{\rm{\Delta }}}_{p}|,|{{\rm{\Delta }}}_{m}|\}\ll \{{\omega }_{m},{\omega }_{d}\}$. *J* = *gα* is the effective nonlinear coupling strength between the optical and mechanical modes. Without loss of generality, *J*, *ε*_*p*_ and *ε*_*m*_ are assumed to be real and the phase difference between the driving fields is denoted by *θ*. For simplicity, we set *δ*_*p*_ = 2*ω*_*d*_ and Δ_*c*_ = 2(*ω*_*m*_ + 2*g*|*α*|^2^ + *g*), then we have *δ* = 0 and Δ ≡ Δ_*m*_ = Δ_*p*_/2, and the effective Hamiltonian ${H}_{{\rm{eff}}}^{\text{'}}$ become time-independent as

6 $$\begin{array}{rcl}{H}_{{\rm{eff}}} & = & 2{\rm{\Delta }}{a}^{\dagger }a+{\rm{\Delta }}{b}^{\dagger }b+J({e}^{-i\theta }a{b}^{\dagger 2}+{e}^{i\theta }{a}^{\dagger }{b}^{2})\\  &  & +({\varepsilon }_{p}{a}^{\dagger }+{\varepsilon }_{m}{b}^{\dagger }+{\rm{H}}{\rm{.c}}.).\end{array}$$

To quantify the statistics of the phonons in the system, we consider the second-order correlation functions in the steady state defined by

7 $${g}_{b}^{(2)}(\tau )\equiv \frac{\langle {b}^{\dagger }(t){b}^{\dagger }(t+\tau )b(t+\tau )b(t)\rangle }{{n}_{b}^{2}},$$

where *n*_*b*_ ≡ 〈*b*^†^(*t*)*b*(*t*)〉 is the mean phonon number. The dynamic behavior of the total open system is described by the master equation for the density matrix *ρ*^44^

8 $$\begin{array}{rcl}\frac{d\rho }{dt} & = & -i[{H}_{{\rm{e}}ff},\rho ]+\frac{{\gamma }_{c}}{2}(2a\rho {a}^{\dagger }-{a}^{\dagger }a\rho -\rho {a}^{\dagger }a)\\  &  & +\frac{{\gamma }_{m}}{2}(2b\rho {b}^{\dagger }-{b}^{\dagger }b\rho -\rho {b}^{\dagger }b)\\  &  & +{\gamma }_{m}{n}_{{\rm{th}}}(b\rho {b}^{\dagger }+{b}^{\dagger }\rho b-{b}^{\dagger }b\rho -\rho b{b}^{\dagger }),\end{array}$$

where we assume that the mean thermal photon number is negligible for the frequency of the optical mode is very high, and *n*_th_ is the mean number of the thermal phonons, given by the Bose-Einstein statistics *n*_th_ = [exp(*ħω*_*m*_/*k*_*B*_*T*) − 1]^−1^ with the Boltzmann constant *k*_*B*_ and the environmental temperature *T*. The second-order correlation function ${g}_{b}^{(2)}(\tau )$ can be calculated by solving the master equation (8) numerically within a truncated Fock space.

It is instructive to find the optimal conditions for strong phonon antibunching before the numerical calculations of the second-order correlation function of the phonons. Following the approach given in ref.^22^, the optimal conditions for UCPNB can be derived analytically with the effective Hamiltonian *H*_eff_ given in equation (6), in the limit *T* → 0 and the weak driving condition $\{{\varepsilon }_{p},{\varepsilon }_{m}\}\ll \{{\gamma }_{c},{\gamma }_{m}\}$. The derivation of the the optimal conditions is provided in the section of Methods. When *θ* = *Nπ* (*N* is an integer), the optimal conditions are simply given by

9 $${{\rm{\Delta }}}_{{\rm{opt}}}=\mathrm{0,}$$

10 $${J}_{{\rm{opt}}}=-\frac{{\varepsilon }_{m}^{2}{\gamma }_{c}}{{\varepsilon }_{p}{\gamma }_{m}\,\cos \,\theta }.$$

When *θ* ≠ *Nπ*, the optimal conditions become

11 $${{\rm{\Delta }}}_{{\rm{opt}}}=\frac{({\gamma }_{c}-2{\gamma }_{m})\cos \,\theta \pm \sqrt{{\rm{\Psi }}}}{8\,\sin \,\theta },$$

12 $${J}_{{\rm{opt}}}=-\frac{{\varepsilon }_{m}^{2}}{{\varepsilon }_{p}}\frac{4{\gamma }_{c}}{({\gamma }_{c}+2{\gamma }_{m})\cos \,\theta \pm \sqrt{{\rm{\Psi }}}},$$

where

13 $${\rm{\Psi }}={(2{\gamma }_{m}-{\gamma }_{c})}^{2}{\cos }^{2}\theta -8{\gamma }_{c}{\gamma }_{m}{\sin }^{2}\theta .$$

In order to make sure that Δ_opt_ and *J*_opt_ given in equations (11) and (12) have real solutions, the phase *θ* should satisfy the condition

14 $$|\theta -N\pi |\le {\tan }^{-1}(\sqrt{\frac{{(2{\gamma }_{m}-{\gamma }_{c})}^{2}}{8{\gamma }_{c}{\gamma }_{m}}}).$$

We take *J*_opt_ > 0 in the following numerical calculations, so that *N* should be an odd number. Without loss of generality, we choose *N* = 1.

### Numerical results

**Figure 2:**
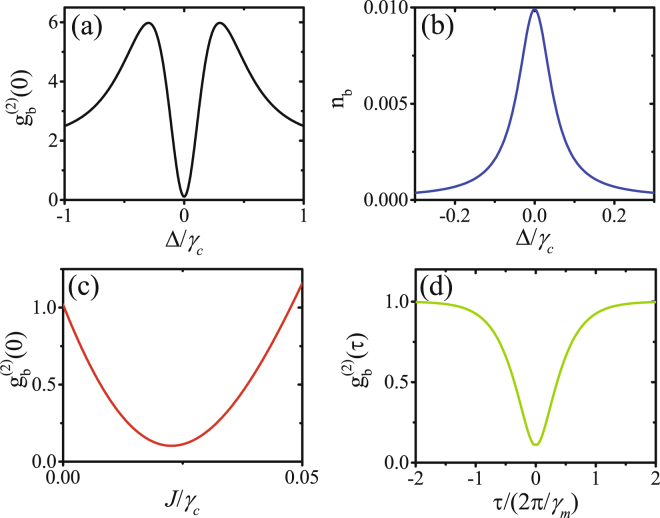
(**a**) ${g}_{b}^{(2)}(0)$ is plotted as a function of the detuning Δ/*γ*_*c*_ with the effective coupling strength *J* = 0.025*γ*_*c*_; (**b**) mean phonon number *n*_*b*_ is plotted as a function of Δ/*γ*_*c*_ with *J* = 0.025*γ*_*c*_; (**c**) ${g}_{b}^{(2)}(0)$ is plotted as a function of *J*/*γ*_*c*_ with Δ = 0; (**d**) ${g}_{b}^{(2)}(\tau )$ is plotted as a function of the normalized time delay *τ*/(2*π*/*γ*_*m*_) with Δ = 0 and *J* = 0.025*γ*_*c*_. The other parameters are *ε*_*m*_ = 0.005*γ*_*c*_, *ε*_*p*_ = 0.01*γ*_*c*_, *θ* = *π*, *γ*_*m*_ = *γ*_*c*_/10, and *n*_th_ = 10^−4^.

In order to confirm the appearing of optimal UCPNB with the optimal parameters given in equations (9–13), we numerically solve the master equation (8) and calculate the second-order correlation functions ${g}_{b}^{(2)}(\tau )$. In Fig. 2(a), the equal-time second-order correlation functions ${g}_{b}^{(2)}(0)$ is plotted as a function of the detuning Δ/*γ*_*c*_ with the effective coupling strength *J* = 0.025*γ*_*c*_ and phase *θ* = *π*. It is clear that the optimal phonon blockade appears at the detuning Δ = 0 and this agrees well with the analytical result given in equation (9). The corresponding mean phonon number *n*_*b*_ is plotted in Fig. 2(b). The maximal value of *n*_*b*_ also appears at the detuning Δ = 0 for resonant driving. The dependence of ${g}_{b}^{(2)}(0)$ on the strength of the effective coupling *J*/*γ*_*c*_ is shown in Fig. 2(c) for Δ = 0 and *θ* = *π*. There is a minimal value of ${g}_{b}^{(2)}(0)$ around *J* ≈ 0.025*γ*_*c*_ which is in agreement with equation (10). ${g}_{b}^{(2)}(\tau )$ is plotted as a function of the normalized time delay *τ*/(2*π*/*γ*_*m*_) in Fig. 2(d) with Δ = 0, *J* = 0.025*γ*_*c*_ and *θ* = *π*. The time duration for strong phonon antibunching is about the lifetime of the phonons.

**Figure 3:**
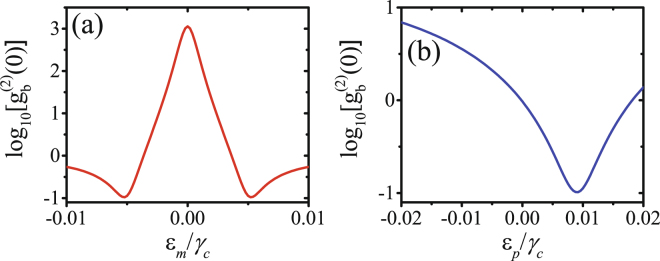
${g}_{b}^{(2)}(0)$ is plotted (**a**) as a function of the mechanical driving strength *ε*_*m*_/*γ*_*c*_ with optical driving strength *ε*_*p*_ = 0.01*γ*_*c*_, (**b**) as a function of the optical driving strength *ε*_*p*_/*γ*_*c*_ with mechanical driving strength *ε*_*m*_ = 0.005*γ*_*c*_. The other parameters are Δ = 0, *J* = 0.025*γ*_*c*_, *θ* = *π*, *γ*_*m*_ = *γ*_*c*_/10, and *n*_th_ = 10^−4^.

There are two weak driving fields applied to the system with the driving strengths *ε*_*p*_ and *ε*_*m*_ and they can allow for dynamic control of the phonon statistics by tuning the strengths and the phase difference of driving fields. In Fig. 3 for *θ* = *π*, ${g}_{b}^{(2)}(0)$ is plotted (a) as a function of the mechanical driving strength *ε*_*m*_/*γ*_*c*_ with optical driving strength *ε*_*p*_ = 0.01*γ*_*c*_, (b) as a function of the optical driving strength *ε*_*p*_/*γ*_*c*_ with mechanical driving strength *ε*_*m*_ = 0.005*γ*_*c*_. The minimal ${g}_{b}^{(2)}(0)$ appears with mechanical driving strength *ε*_*m*_ ≈ ±0.005*γ*_*c*_ in Fig. 3(a) and with optical driving strength *ε*_*p*_ ≈ 0.01*γ*_*c*_ in Fig. 3(b). These results are consistent with the analytically expression given in equation (10). Moreover, as shown in Fig. 3(a), the phonons exhibit strong bunching as *ε*_*m*_ = 0 but exhibit strong antibunching as *ε*_*m*_ = 0.005*γ*_*c*_. These phenomena can be understand as follows: when *ε*_*m*_ = 0, phonons only can be generated in pairs by the optical driving field, so the phonons exhibit strong bunching; when *ε*_*m*_ ≠ 0, phonons pairs can be generated in two different ways (by optical driving field or by mechanical driving field), the strong phonon antibunching is induced by the destructive interference between the two different ways for phonon pairs generation when *ε*_*m*_ ≈ *ε*_*p*_/2 = 0.005*γ*_*c*_. As shown in Fig. 3(b), the phonons exhibit strong antibunching as *ε*_*p*_ = 0.01*γ*_*c*_ but exhibit bunching as *ε*_*p*_ > 0.02*γ*_*c*_ or *ε*_*p*_ < 0. So we can control the phonon statistics dynamically by tuning the strengths of driving fields.

**Figure 4:**
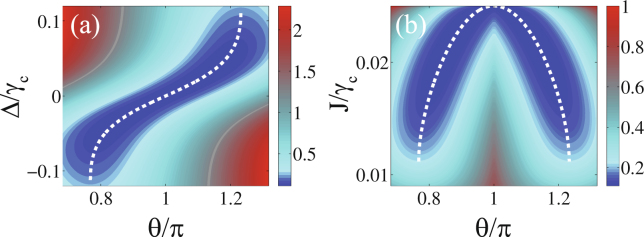
(**a**) Contour plot of ${g}_{b}^{(2)}(0)$ as a function of the phase *θ*/*π* and the detuning Δ/*γ*_*c*_ for effective coupling strength *J* given in equation (19); (**b**) contour plot of ${g}_{b}^{(2)}(0)$ as a function of *θ*/*π* and *J*/*γ*_*c*_ for Δ given in equation (17). The white dashed lines refer to equation (11) in (**a**) and refer to equation (12) in (**b**). The other parameters are *ε*_*m*_ = 0.005*γ*_*c*_, *ε*_*p*_ = 0.01*γ*_*c*_, *γ*_*m*_ = *γ*_*c*_/10, and *n*_th_ = 10^−4^.

In Fig. 4(a), we show the contour plot of ${g}_{b}^{(2)}(0)$ as a function of the phase *θ*/*π* and the detuning Δ/*γ*_*c*_ with the effective coupling strength *J* given by

15 $$J=-\frac{{\varepsilon }_{m}^{2}}{{\varepsilon }_{p}}\frac{{\gamma }_{c}}{{\gamma }_{m}\,\cos \,{\theta }_{c}+2{\rm{\Delta }}\,\sin \,{\theta }_{c}},$$

where

16 $${\theta }_{c}=\pi +{\tan }^{-1}[\frac{2{\rm{\Delta }}({\gamma }_{c}-2{\gamma }_{m})}{8{{\rm{\Delta }}}^{2}+{\gamma }_{c}{\gamma }_{m}}].$$

In Fig. 4(b), we show the contour plot of ${g}_{b}^{(2)}(0)$ as a function of *θ*/*π* and *J*/*γ*_*c*_ with the detuning Δ given by

17 $${\rm{\Delta }}=\frac{{\varepsilon }_{p}J{\gamma }_{m}\,\sin \,{\theta }_{c}}{4{\varepsilon }_{m}^{2}+2{\varepsilon }_{p}J\,\cos \,{\theta }_{c}},$$

where

18 $${\theta }_{c}=\{\begin{array}{cc}{\cos }^{-1}[-\frac{{\varepsilon }_{p}^{2}{J}^{2}{\gamma }_{m}+2{\varepsilon }_{m}^{4}{\gamma }_{c}}{{\varepsilon }_{m}^{2}{\varepsilon }_{p}J(2{\gamma }_{m}+{\gamma }_{c})}] & \pi \mathrm{/2} < \theta  < \pi ,\\ 2\pi -{\cos }^{-1}[-\frac{{\varepsilon }_{p}^{2}{J}^{2}{\gamma }_{m}+2{\varepsilon }_{m}^{4}{\gamma }_{c}}{{\varepsilon }_{m}^{2}{\varepsilon }_{p}J(2{\gamma }_{m}+{\gamma }_{c})}] & \pi  < \theta  < 3\pi \mathrm{/2}.\end{array}$$

The white dashed lines refer to equation (11) in Fig. 4(a) and refer to equation equation (12) in Fig. 4(b). The white dashed lines conform very closely to optimal region (dark blue region) for phonon antibunching. Obviously, the phonon statistic properties are also dependent on the phase difference *θ* of the driving fields.

**Figure 5:**
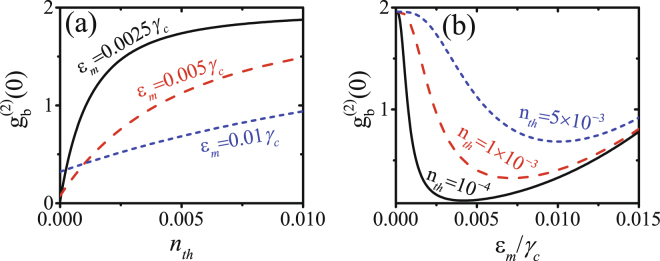
(**a**) ${g}_{b}^{(2)}(0)$ is plotted as a function of the mean thermal phonon number *n*_th_ with different driving strengths *ε*_*m*_/*γ*_*c*_, (**b**) ${g}_{b}^{(2)}(0)$ is plotted as a function of the driving strength *ε*_*m*_/*γ*_*c*_ with different mean thermal phonon number *n*_th_. The other parameters are Δ = 0, *J* = 0.025*γ*_*c*_, *θ* = *π*, ${\varepsilon }_{p}={\varepsilon }_{m}^{2}\mathrm{/(0.0025}{\gamma }_{c})$ and *γ*_*m*_ = *γ*_*c*_/10.

Different from the photon blockade in optical cavities with frequency 10^14^ Hz, where the mean thermal photon number is negligible, the effect of the thermal phonons should be considered in the investigation of phonon blockade in mechanical resonators even with microwave-frequency^13^. In Fig. 5(a), ${g}_{b}^{(2)}(0)$ is plotted as a function of the mean thermal phonon number *n*_th_. One can see that the phonon antibunching becomes weaker with the increase of the the mean thermal phonon number *n*_th_. In Fig. 5(b), ${g}_{b}^{(2)}(0)$ is plotted as a function of the driving strength *ε*_*m*_/*γ*_*c*_ with different mean thermal phonon number *n*_th_. The optimal phonon blockade can be obtained by properly increasing the driving strengths according to the mean thermal phonon number *n*_th_.

## Discussion

In summary, we have investigated the UCPNB in a quadratically coupled optomechanical system. It has been shown that strong phonon antibunching can be observed even with weak effective second-order nonlinear interaction. The optimal conditions for UCPNB were given analytically and they well coincided with the numerical results. Moreover, the phonon statistics can be dynamically controlled by tuning the strengths and the phase difference of external driving fields. The results show that tunable single-phonon sources can be realize in the quadratically coupled optomechanical systems.

Based on the numerical results, we can estimate the experimental parameters for realizing our proposal. For instance, if we take the parameters according to the numerical simulations in ref.^35^, *ω*_*m*_/2*π* = 225 MHz, *γ*_*c*_/2*π* = 20 MHz, *g*/2*π* = 10 kHz, and *γ*_*m*_/2*π* = 80 kHz, then the effective coupling strength *J* = 0.025*γ*_*c*_ can be realized with |*α*| = 50 when the strength of the strong optical driving field is taken as Ω_*L*_ ≈ 27.5 GHz. In order to reduce the negative impact of the environment on the phonon statistics, the experiments should be done under low temperature with high mechanical frequency. The mechanical resonators with frequency above 5 GHz have already be realized in many groups^45,46^, and the mean thermal phonon number will be smaller than 10^−4^ at a temperature of 25 mK in a dilution refrigerator. So far as we know, the second-order correlation of phonons can not be observed directly. In a recent experiment, the correlations of phonons have been observed indirectly by coupling an auxiliary optical cavity to the mechanical resonator and measuring photon correlations of the output field from the optical cavity^47^.

## Methods

In this section, we will derive the optical conditions for UCPNB analytically with the effective Hamiltonian *H*_eff_ given in equation (6), in the limit *T* → 0 and the weak driving condition $\{{\varepsilon }_{p},{\varepsilon }_{m}\}\ll \{{\gamma }_{c},{\gamma }_{m}\}$. The wave function can be expanded on a Fock state basis as

19 $$|\psi \rangle ={C}_{00}|\mathrm{0,0}\rangle +{C}_{10}|\mathrm{1,0}\rangle +{C}_{01}|\mathrm{0,1}\rangle +{C}_{02}|\mathrm{0,2}\rangle +\cdots ,$$

where $|n,m\rangle$ represents the state with *n* photons and *m* phonons, and the corresponding coefficient |*C*_*nm*_|^2^ denotes the occupying probability. In the weak driving condition, i.e. $\{{\varepsilon }_{p},{\varepsilon }_{m}\}\ll \{{\gamma }_{c},{\gamma }_{m}\}$, we will have $|{C}_{00}|\gg \{|{C}_{10}|,|{C}_{01}|,|{C}_{02}|\}\gg \{|{C}_{11}|,|{C}_{02}|,|{C}_{12}|\}\gg \cdots$, so the wave function can be truncated to the one-photon and two-phonon states approximately. Substituting the wave function in equation (19) and the Hamiltonian in equation (6) into the Schrödinger’s equation $id|\psi \rangle /dt={H}_{{\rm{eff}}}|\psi \rangle$, then the dynamical equations for the coefficients *C*_*nm*_ are shown as

20 $$\frac{d}{dt}{C}_{10}=-(\frac{{\gamma }_{c}}{2}+i2{\rm{\Delta }}){C}_{10}-i{\varepsilon }_{p}{C}_{00}-i\sqrt{2}J{e}^{i\theta }{C}_{02},$$

21 $$\frac{d}{dt}{C}_{01}=-(\frac{{\gamma }_{m}}{2}+i{\rm{\Delta }}){C}_{01}-i{\varepsilon }_{m}{C}_{00},$$

22 $$\frac{d}{dt}{C}_{02}=-({\gamma }_{m}+i2{\rm{\Delta }}){C}_{02}-i\sqrt{2}{\varepsilon }_{m}{C}_{01}-i\sqrt{2}J{e}^{-i\theta }{C}_{10}.$$

In the steady state, i.e. *dC*_*nm*_/*dt* = 0, the phonon blockade ${g}_{b}^{\mathrm{(2)}}\mathrm{(0)}\approx 0$ appears when *C*_02_ ≈ 0. Under the condition for phonon blockade, i.e. *C*_02_ ≈ 0, the coefficients *C*_10_, *C*_01_ and *C*_00_ satisfy the linear equations

23 $$0=-(\frac{{\gamma }_{c}}{2}+i2{\rm{\Delta }}){C}_{10}-i{\varepsilon }_{p}{C}_{00},$$

24 $$0=-(\frac{{\gamma }_{m}}{2}+i{\rm{\Delta }}){C}_{01}-i{\varepsilon }_{m}{C}_{00},$$

25 $$0=-i\sqrt{2}{\varepsilon }_{m}{C}_{01}-i\sqrt{2}J{e}^{-i\theta }{C}_{10}.$$

From equations (23) and (24), *C*_10_ and *C*_01_ are given by

26 $${C}_{10}=\frac{-i2{\varepsilon }_{p}}{{\gamma }_{c}+i4{\rm{\Delta }}}{C}_{00},$$

27 $${C}_{01}=\frac{-i2{\varepsilon }_{m}}{{\gamma }_{m}+i2{\rm{\Delta }}}{C}_{00}.$$

Substituting *C*_10_ and *C*_01_ into equation (25), we obtain

28 $$0=(\frac{{\varepsilon }_{m}^{2}}{{\gamma }_{m}+i2{\rm{\Delta }}}+\frac{{\varepsilon }_{p}J{e}^{-i\theta }}{{\gamma }_{c}+i4{\rm{\Delta }}}){C}_{00}.$$

As |*C*_00_| ≈ 1 ≠ 0, then we get the conditions for the optimal parameters *J*_opt_ and Δ_opt_ as

29 $${\varepsilon }_{p}{J}_{{\rm{opt}}}({\gamma }_{m}\,\cos \,\theta +2{{\rm{\Delta }}}_{{\rm{opt}}}\,\sin \,\theta )+{\varepsilon }_{m}^{2}{\gamma }_{c}=\mathrm{0,}$$

30 $${\varepsilon }_{p}{J}_{{\rm{opt}}}(2{{\rm{\Delta }}}_{{\rm{opt}}}\,\cos \,\theta -{\gamma }_{m}\,\sin \,\theta )+4{\varepsilon }_{m}^{2}{{\rm{\Delta }}}_{{\rm{opt}}}=0.$$

The optimal parameters for phonon blockade given in equations (9–13) are obtained by solving the equations (29) and (30).
